# A Facile One-Step Synthesis of Cuprous Oxide/Silver Nanocomposites as Efficient Electrode-Modifying Materials for Nonenzyme Hydrogen Peroxide Sensor

**DOI:** 10.3390/nano9040523

**Published:** 2019-04-03

**Authors:** Kaixiang Yang, Zhengguang Yan, Lin Ma, Yiping Du, Bo Peng, Jicun Feng

**Affiliations:** 1 Institute of Microstructure and Property of Advanced Materials, Beijing University of Technology, Beijing 100124, China; ykx233210@gmail.com (K.Y.); hokingma@gmail.com (L.M.); duyp@emails.bjut.edu.cn (Y.D.); pengbo2017@emails.bjut.edu.cn (B.P.); fengjc0619@163.com (J.F.); 2 Beijing Key Laboratory of Microstructure and Properties of Solids, Beijing University of Technology, Beijing 100124, China

**Keywords:** cuprous oxide/silver, nanocomposites, hydrogen peroxide, electrochemistry, sensor

## Abstract

Cuprous oxide/silver (Cu_2_O/Ag) nanocomposites were prepared via a facile one-step method and used to construct an electrochemical sensor for hydrogen peroxide (H_2_O_2_) detection. In this method, AgNO_3_ and Cu(NO_3_)_2_ were reduced to Cu_2_O/Ag nanocomposites by glucose in the presence of hexadecyl trimethyl ammonium bromide (CTAB) at a low temperature. The optimum condition was the molar ratio of silver nitrate and copper nitrate of 1:10, the temperature of 50 °C. Under this condition, Cu_2_O/Ag nanocomposites were obtained with uniformly distributed and tightly combined Cu_2_O and Ag nanoparticles. The size of Cu_2_O particles was less than 100 nm and that of Ag particles was less than 20 nm. Electrochemical experiments indicate that the Cu_2_O/Ag nanocomposites-based sensor possesses an excellent performance toward H_2_O_2_, showing a linear range of 0.2 to 4000 μM, a high sensitivity of 87.0 μA mM^−1^ cm^−2^, and a low detection limit of 0.2 μM. The anti-interference capability experiments indicate this sensor has good selectivity toward H_2_O_2_. Additionally, the H_2_O_2_ recovery tests of the sensor in diluted milk solution signify its potential application in routine H_2_O_2_ analysis.

## 1. Introduction

The rapid and sensitive detection of H_2_O_2_ has attracted a lot of attention because of the applications of H_2_O_2_ in food [1], medicine [2], chemical industry [3], and environmental protection [4] as a common intermediate and oxidant, as well as its involvement in many biological events and intracellular pathways [5]. Conventional techniques for H_2_O_2_ determination have been developed, such as titrimetry [6], colorimetry [7], chemiluminescence [8], fluorescence resonance energy transfer-based upconversion [9], chromatography [10], and electrochemical methods [11]. Among these techniques, the electrochemical method is considered to be a prospective approach for its good selectivity, high sensitivity, and simple manipulation [4]. Although enzyme-based H_2_O_2_ sensors exhibit prominent advantages of high selectivity, the complexity of the enzyme curing process and instability to toxic chemicals limit their practical applications [12]. Therefore, a growing interest in developing enzyme-free sensors for detecting H_2_O_2_ has been aroused in this field [13,14]. Catalytic active nanomaterials, including noble metals [15], transition metal oxides [16], and other transition metal compounds [17,18], thanks to their selectivity and high activity, have been widely used to construct nonenzyme H_2_O_2_ sensors.

In recent years, as a typical transition metal oxide, cuprous oxide (Cu_2_O) has attracted increasing attention as a promising candidate for H_2_O_2_ sensors due to its proper redox potentials, easy production process, and low cost [19,20]. Unfortunately, pristine Cu_2_O sensors demonstrate low sensitivity and narrow linear detection ranges [21,22]. Combination with other materials to prepare composites is one effective way to improve the performance of Cu_2_O-based H_2_O_2_ sensors. The metal nanoparticles, thanks to their good conductivity and high electrocatalytic activity, could largely facilitate the electron transfer on the surface of transition-metal oxides and improve their electrocatalytic activity [23]. Up to now, different metal particles have been introduced to transition-metal oxides for H_2_O_2_ sensors, such as Au/MnO_2_ [24], Au/Fe_3_O_4_ [25], Ag/MnO_2_/MWCNTs [26], Au/Cu_2_O [27], and Pt/Fe_3_O_4_/Graphene [28]. Particularly, Ag nanoparticles (AgNPs) exhibit higher conductivity and lower cost compared with Au and Pt, and could produce synergistic effects when combined with some metal oxides [26], thus they are a promising material for improving the catalytic performance of the transition-metal oxides. Therefore, it is promising to introduce Ag into Cu_2_O-based composites to fabricate H_2_O_2_ sensors.

Although these transition-metal oxide/metal nanocomposites mentioned above do fairly well in H_2_O_2_ sensing, the preparation of these materials is usually complicated, multistep, and time-consuming. The conventional routes would synthesize metal oxides first, and then modify metal particle to the surface of metal oxides. Therefore, it makes sense to simplify the synthesis steps for material preparation.

In this work, we introduced a facile one-step procedure to combine Cu_2_O with Ag to prepare Cu_2_O/Ag nanocomposites. The effects of experimental conditions on composition and morphology of the nanocomposites were studied. The electrochemical measurements were applied to elucidate the sensing application of Cu_2_O/Ag nanocomposites, and the anti-interference capability experiments and the H_2_O_2_ recovery tests indicate Cu_2_O/Ag nanocomposites could be a promising material for H_2_O_2_ detection.

## 2. Materials and Methods

### 2.1. Reagents and Chemicals

All reagents were of analytical reagent grade and used without further purification. Cu(NO_3_)_2_·3H_2_O, AgNO_3_, hexadecyl trimethyl ammonium bromide (CTAB), and ethanol were purchased from Beijing Chemical Reagents Company (Beijing, China). *D*-glucose, NaOH, urea, fructose, *L*-ascorbic acid, Na_2_HPO_4_, and H_2_O_2_ solution (30%) were purchased from Tianjin Fuchen Chemical Reagent Co, (Tianjin, China). Ltd. K_3_[Fe(CN)_6_] and NaH_2_PO_4_·12H_2_O were purchased from Aladdin Reagent Co (Shanghai, China). All aqueous solutions were prepared with double-distilled water.

### 2.2. Synthesis of Cu_2_O/Ag Nanocomposites and Modification of Electrode

**Figure 1:**
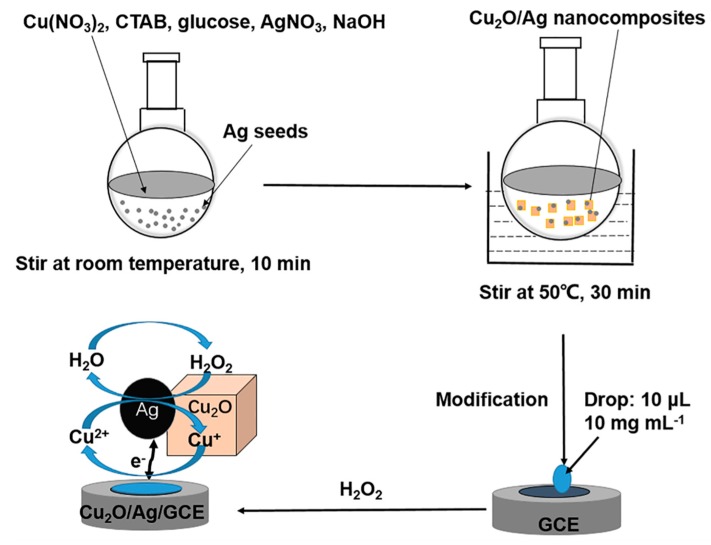
Schematic illustration for the facile method to prepare Cu_2_O/Ag/GCE.

The preparation of Cu_2_O/Ag nanocomposites was carried out in aqueous solution using glucose as reducing agent and CTAB as dispersing agent. A typical procedure is performed as illustrated in Figure 1. A 0.035 g portion of AgNO_3_ (0.2 mmol) dissolved in 20 mL double-distilled water was marked as solution A. Next, 0.5 g Cu(NO_3_)_2_·3H_2_O (2 mmol) and 0.5 g glucose (2.5 mmol) were dissolved in 50 mL double-distilled water, and then 10 mL aqueous solution of CTAB (0.014 mol L^−1^) was added into the mixture under stirring. The solution was marked as solution B. The molar ratios of AgNO_3_ and Cu(NO_3_)_2_ could be varied by changing the quantity of AgNO_3_ according to the requirement. A 0.5 g portion of NaOH (12.5 mmol) dissolved in 20 mL double-distilled water was marked as solution C. The solutions A (20 mL), B (60 mL), and C (20 mL) were added into a flask under stirring at room temperature. The solution was stirred for another 10 min and a gray precipitate formed. Then the reaction suspension was heated under vigorous stirring (500 rpm) at a temperature of 50 °C for 30 min and the mixture turned brown-gray gradually. Finally, the product was separated by centrifugation and washed with water and ethanol for three times. The amount of ethanol and water used to wash the products was 20 mL per 100 mg each time, respectively. The products were dried at 70 °C overnight. Note, it is important to recover any organic solvent to reduce the environmental burden and improve the sustainability of the methodology [29]. The alcohol used to wash the products could be recovered by fractionation for secondary use.

A glassy carbon electrode (GCE) was polished, cleaned, and dried for the fabrication of the sensor. Generally, 10 mg of Cu_2_O/Ag nanocomposites were dispersed into 1 mL double-distilled water and sonicated for 15 min. A 10 µL portion of the suspension was dropped onto the GCE and then dried in air at room temperature. The modified electrode was marked as Cu_2_O/Ag/GCE. The Cu_2_O sample without Ag was used similarly to modify the electrode, which was marked as Cu_2_O/GCE.

### 2.3. Electrochemical Experiments

Electrochemical measurements were carried out with a PARSTAT 2273 potentiostat galvanostat (Princeton Applied Research, Oak Ridge, TN, USA) in a three-electrode system, with the modified GCE (0.3 cm in diameter) as working electrode, Ag/AgCl/KCl (sat.) as reference electrode, and a platinum sheet as the counter electrode. The cyclic voltammetry profiles (CVs) and current–time profiles were measured in an N_2_-saturated PBS solution (0.1 M, pH = 7.2) at room temperature. The electrochemical impedance spectroscopy (EIS) was tested in a 5 mM [Fe(CN_6_)^3−^] solution containing 0.1 M KCl with a frequency range of 10^−2^–10^5^ Hz and an amplitude of 10 mV.

### 2.4. Material Characterization Techniques

The powder X-ray diffraction (XRD) patterns of the as-prepared materials were carried out on a D8 Advance X-ray diffractometer (Bruker AXS GmbH, Karlsruhe, Germany) with Cu Kα radiation (λ = 1.54178 Å). The scanning electron microscopy (SEM) images of the products were characterized using an FEI Quanta 600 field emission scanning electron microscope (FEI Company, Hillsboro, OR, USA). The transmission electron microscopy (TEM) images and electron diffraction (ED) patterns were obtained using an FEI T20 transmission electron microscope (FEI Company, Hillsboro, OR, USA) working at 180 kV. High resolution transmission electron microscopy (HRTEM) images and electron dispersive spectra mapping of the materials (EDS mapping) were obtained using an FEI Titan G2 spherical-aberration-corrected transmission electron microscope (FEI Company, Hillsboro, OR, USA) working at 200 kV. The X-ray photoelectron spectra (XPS) of materials were characterized by an ESCALAB 250Xi X-ray Photoelectron Spectrometer (Thermo Fisher Scientific, Waltham, MA, USA) with a monochromatic Al Kα X-ray and a 500 μm nominal spot size, and the high-resolution scans were collected with a pass energy of 30 eV and a step size of 0.05 eV.

## 3. Results and Discussion

### 3.1. Effect of Experimental Conditions on Composition and Morphology

**Figure 2:**
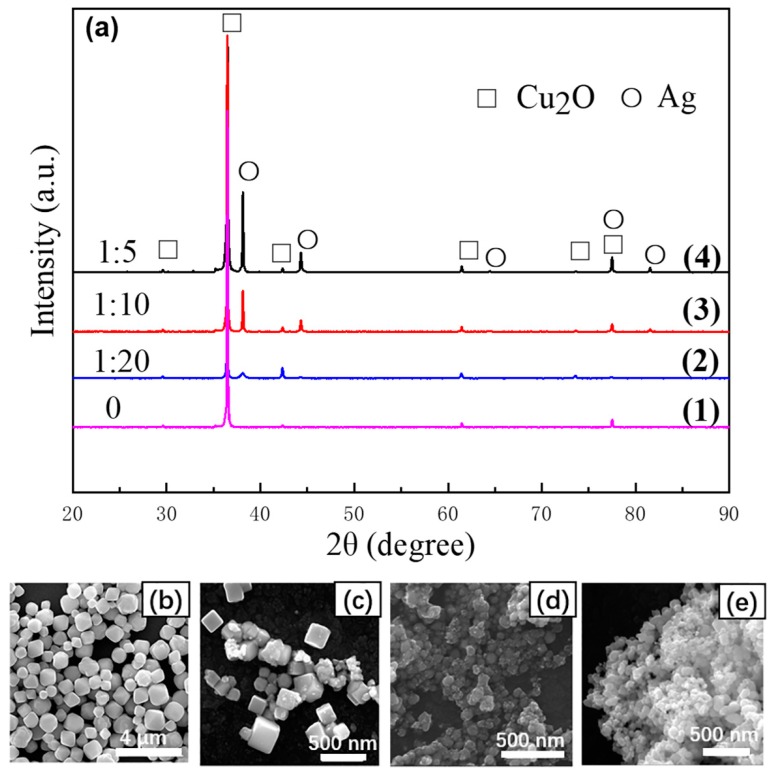
(**a**) XRD patterns of the as-synthesized nanomaterials prepared in different molar ratio of AgNO_3_:Cu(NO_3_)_2_. *n*_AgNO3_:*n*_Cu(NO3)2_ = 0 (**1**), 1:20 (**2**), 1:10 (**3**), and 1:5 (**4**), respectively. SEM images of nanomaterials prepared at different molar ratio of AgNO_3_:Cu(NO_3_)_2_. *n*_AgNO3_:*n*_Cu(NO3)2_ = 0 (**b**), 1:20 (**c**), 1:10 (**d**), 1:5 (**e**), respectively.

In this study, a simple one-step method was used to prepare Cu_2_O/Ag nanocomposites successfully. The dose of Cu(NO_3_)_2_ 0.5 g (2 mmol) was kept unchanged, and the dose of AgNO_3_ was changed. Different molar ratios of AgNO_3_ and Cu(NO_3_)_2_ in the reactants (*n*_AgNO3_:*n*_Cu(NO3)2_ = 0, 1:20, 1:10, 1:5, respectively) were used to prepare nanomaterials with different compositions at the temperature of 50 °C. The XRD patterns of these nanocomposites prepared with different molar ratios of AgNO_3_ and Cu(NO_3_)_2_ are shown in Figure 2a, from which we can easily find that all the nanocomposites show the strong diffraction peaks of the cubic crystal structure of the Cu_2_O phase (space group: *Pn3m*, JCPDS 5-667 [30]) with fitted lattice parameter of *a* = 0.430 nm. The six peaks (square notations) with 2θ values of 29.68, 36.50, 42.40, 61.52, 73.70, and 77.57 were observed and could be assigned to diffraction from the (110), (111), (200), (220), (311), and (222) planes, respectively. In addition, the XRD pattern of products (*n*_AgNO3_:*n*_Cu(NO3)2_ = 1:5, 1:10, 1:20, respectively) showed extra peaks (round notations) because of the introduction of Ag, and the XRD peaks at 2θ degrees of 38.11, 44.28, 64.43, 77.47, and 81.54 can be attributed to the (111), (200), (220), (311), and (222) crystalline planes of the face-centered-cubic (fcc) crystalline structure of Ag, respectively (space group: *Fm-3m*, JCPDS 4-783 [31]) with fitted lattice parameter of *a* = 0.409 nm. In addition, with the molar ratio of *n*_AgNO3_:*n*_Cu(NO3)2_ decreased, the intensity of the Ag peaks decreased obviously, which indicated that the Ag content in the nanocomposites was positively correlated with the amount of AgNO_3_ added.

The SEM was used to investigate the morphology of nanomaterials prepared with different molar ratios of AgNO_3_ and Cu(NO_3_)_2_ under the temperature of 50 °C, as is shown in Figure 2b–e, from which we can easily find that the size of Cu_2_O particles decreased obviously with the increase of molar ratio of AgNO_3_:Cu(NO_3_)_2_. The average particle size of pure Cu_2_O prepared without addition of AgNO_3_ was between 400 nm and 1.2 μm (see the size distribution histograms shown in Figure S1a, SI). However, when *n*_AgNO3_:*n*_Cu(NO3)2_ = 1:20, Cu_2_O particles of the nanocomposites became much smaller in size (50–300 nm) compared with the pure Cu_2_O prepared; the size distribution histogram is in Figure S1b. The reason for the decrease in sizes for Cu_2_O particles is that a lot of Ag nanoparticles were formed and acted as seeds before the Cu_2_O nanoparticles appeared, which could be observed when the mixture quickly turned gray at room temperature in the process of synthesis. As shown in Figure S2, the size of Ag nanoparticles initially formed was smaller than 20 nm, and they would act as nucleation seeds for Cu_2_O to nucleate on and grow. Therefore, the Cu_2_O particles and Ag particles would form good contact in the step. Then, Cu_2_O particles became small-sized because of these large numbers of Ag seeds. In addition, it can be seen from the SEM images in Figure 2d,e that the size of Cu_2_O became very small (<100 nm) when *n*_AgNO3_:*n*_Cu(NO3)2_ = 1:10 and 1:5. However, when *n*_AgNO3_:*n*_Cu(NO3)2_ = 1:5, the nanoparticles tended to agglomerate. Considering the uniformity of particle size and the dispersion of nanocomposites, 1:10 is the appropriate dosage ratio to prepare Cu_2_O/Ag nanocomposites.

The formation of nanocomposites was also influenced by the reaction temperature. From the XRD patterns in Figure S3, we can easily find that the reaction temperature plays an important role in the formation of Cu_2_O/Ag nanocomposites. At room temperature, only Ag was produced. In contrast, Cu(NO_3_)_2_ was partially reduced to Cu when the temperature was 70 °C, and a mixture of Cu and Ag was synthesized when the temperature raised to 100 °C. Only when the reaction temperature was around 50 °C were Cu_2_O/Ag nanocomposites synthesized.

**Figure 3:**
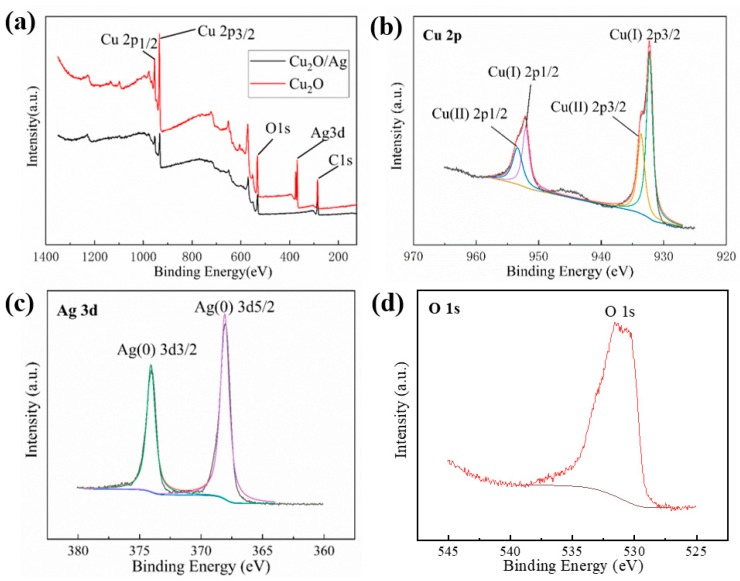
(**a**) XPS survey spectrum of the as-synthesized pure Cu_2_O and Cu_2_O/Ag nanocomposites obtained with *n*_AgNO3_:*n*_Cu(NO3)2_ = 1:10. (**b**) Cu 2p regions of Cu_2_O/Ag nanocomposites. (**c**) Ag 3d regions of the Cu_2_O/Ag nanocomposites. (**d**) O 1s regions of Cu_2_O/Ag nanocomposites.

Additionally, the XPS measurement for the pure Cu_2_O and Cu_2_O/Ag nanocomposites (*n*_AgNO3_:*n*_Cu(NO3)2_ = 1:10) was further carried out to elucidate the valence states of the Cu and Ag element. Figure 3a shows the XPS survey spectra of pure Cu_2_O and Cu_2_O/Ag nanocomposites. The C, Cu, and O elements were detected for both samples [32,33], and the survey spectrum of Cu_2_O/Ag nanocomposites (red line) shows extra peaks which can be assigned to the AgNPs [34]. Figure 3b shows the XPS spectra in Cu 2p regions of the Cu_2_O/Ag nanocomposite, which indicate the existence of Cu_2_O (932.3 eV: Cu(I) 2p_3/2_, 952.1 eV: Cu(I) 2p_1/2_ of Cu_2_O) and the surface of Cu_2_O nanoparticles was slightly oxidized (933.6 eV: Cu(II) 2p_3/2_, 953.4 eV: Cu(II) 2p_1/2_). Figure 3c shows the Ag 3d region of Cu_2_O/Ag nanocomposites with doublet peaks at 374.5 eV and 368.3 eV, which were assigned to the Ag 3d_3/2_ and Ag 3d_5/2_ of Ag(0), respectively. Figure 3d shows the O 1s regions of the Cu_2_O/Ag nanocomposites. The O 1s peak is around 529.7–532.4 eV, which is consistent with the O peak of Cu_2_O reported [33]. We can see clearly from the XPS data above that the AgNPs was introduced to Cu_2_O/Ag nanocomposites successfully.

**Figure 4:**
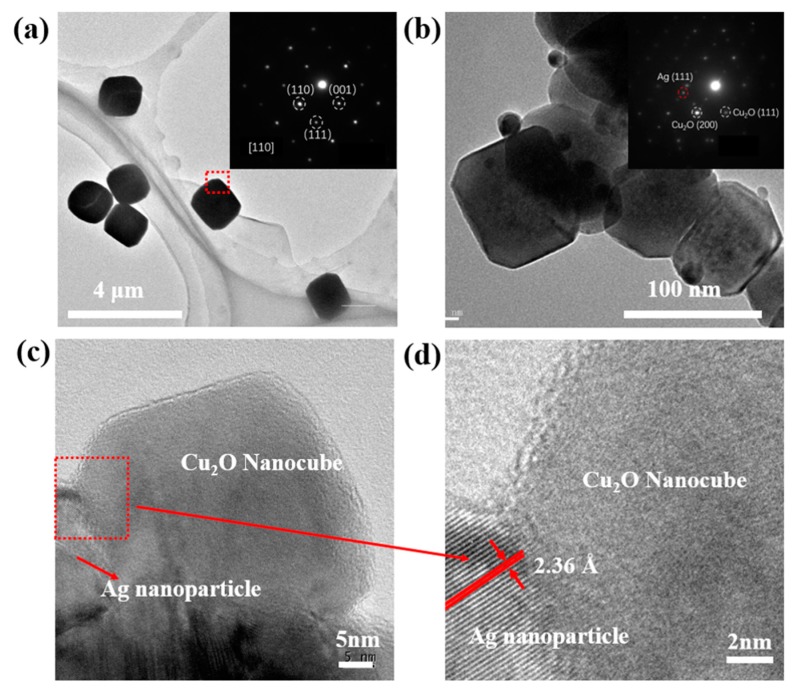
TEM images of the pure Cu_2_O particles and the Cu_2_O/Ag nanocomposites obtained with *n*_AgNO3_:*n*_Cu(NO3)2_ = 1:10. (**a**) The TEM image of Cu_2_O particles (Inset: the SAED pattern of pure Cu_2_O particles); (**b**) The TEM image of Cu_2_O/Ag nanocomposites (Inset: the SAED pattern of Cu_2_O/Ag nanocomposites); (**c**) HRTEM images of Cu_2_O/Ag nanocomposites; (**d**) Enlarged HRTEM image of rectangular region of (**c**).

Figure 4a shows the TEM image and selected-area electron diffraction (SAED) image of pure Cu_2_O particles. The SAED patterns were taken at the edge of the particle and demonstrate a typical fcc structure of Cu_2_O crystals which are of highly crystalline nature [35]. Figure 4b shows the TEM image and SAED pattern of the Cu_2_O/Ag nanocomposites. It can be seen clearly from the TEM image that the size of AgNPs in the nanocomposites is smaller than 20 nm. Meanwhile, the size of Cu_2_O nanocubes is smaller than 100 nm, which is about less than 1/10 the size of the pure Cu_2_O cubes prepared by the same way (Figure S1a). Figure 4c,d are HRTEM images of the Cu_2_O/Ag nanocomposites. The lattice fringes in the particle in Figure 4d are separated by 0.236 nm, in good agreement with the (111) lattice spacing of Ag. In addition, it can be seen clearly that Ag particles are closely attached to Cu_2_O cubes from the HRTEM images.

**Figure 5:**
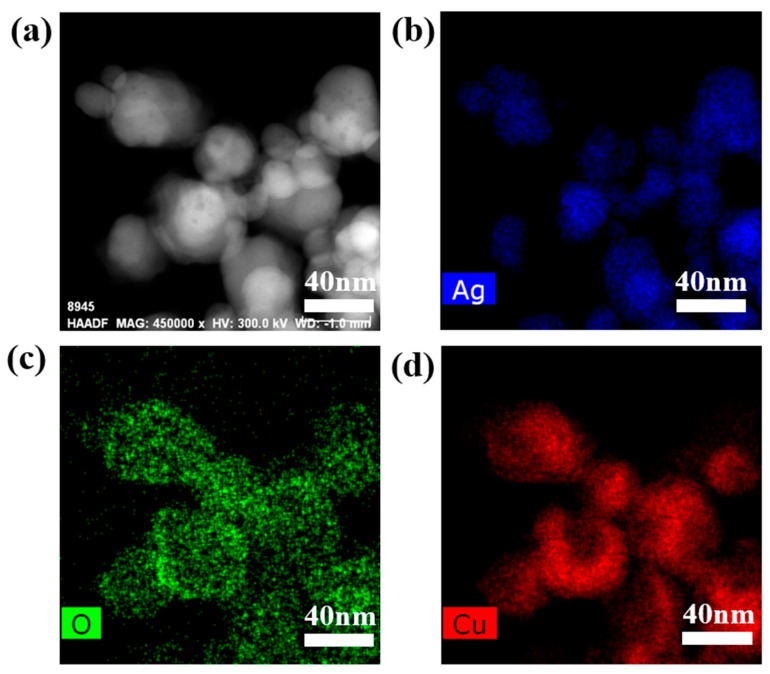
The images of the Cu_2_O/Ag sample obtained with *n*_AgNO3_:*n*_Cu(NO3)2_ = 1:10. (**a**) A scanning transmission microscopy image, and (**b**–**d**) the corresponding EDS mapping images: (**b**) Ag element, (**c**) O element, (**d**) Cu element.

To further observe the combination of Ag and Cu_2_O, EDS mapping was employed as shown in Figure 5. The EDS mapping images confirmed the coexistence of Ag, Cu, and O elements in the Cu_2_O/Ag nanocomposites and further confirmed that the composite material is not a simple mixture of Ag particles and Cu_2_O particles, but a nanoscale composite which is tightly bound together.

### 3.2. Electrochemical Sensing Performances of the Cu_2_O/Ag/GCE for H_2_O_2_ Detection

**Figure 6:**
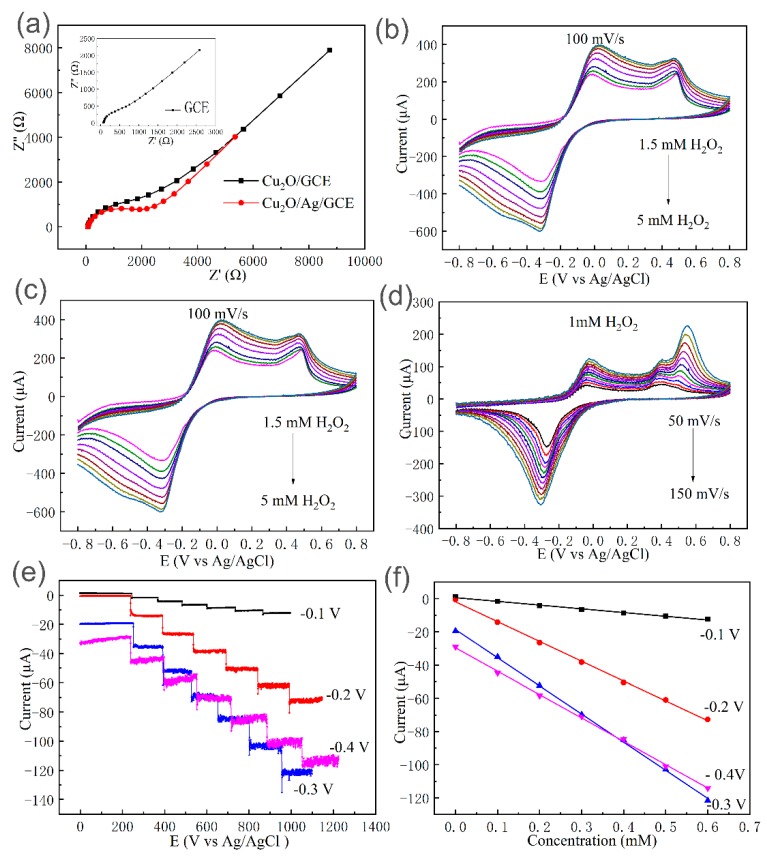
(**a**) Electrochemical impedance plots (Nyquist plots) of Cu_2_O/GCE and Cu_2_O/Ag/GCE in 5 mM [Fe(CN_6_)^3^^−^] containing 0.1 M KCl (Inset: Nyquist plots of bare GCE). (**b**) CVs of bare GCE, Cu_2_O/GCE, and Cu_2_O/Ag/GCE in N_2_-saturated 0.1 M PBS (pH 7.2) in the presence of 1.0 mM H_2_O_2_ at a scan rate of 100 mV/s. (**c**) CVs of Cu_2_O/Ag/GCE in N_2_-saturated 0.1 M PBS (pH 7.2) at a scan rate of 100 mV s^−1^ in the presence of H_2_O_2_ with different concentrations of 1.5, 2.0, 2.5, 3.0, 3.5, 4.0, 4.5, and 5.0 mM. (**d**) CVs of Cu_2_O/Ag/GCE in N_2_-saturated 0.1 M PBS (pH 7.2) containing 1.0 mM H_2_O_2_ at different scan rates (50, 60, 70, 80, 90, 100, 110, 120, 130, 140, and 150 mV s^−1^). (**e**) Current–time curves of the Cu_2_O/Ag/GCE upon successive addition of 0.1 mM H_2_O_2_ into N_2_-saturated 0.1 M PBS (pH = 7.2) under different applied potential of −0.10, −0.20, −0.30, and −0.40 V (vs. Ag/AgCl). (**f**) The corresponding calibration curves of currents vs. H_2_O_2_ concentrations under different potentials (−0.10, −0.20, −0.30, −0.40 V).

The Cu_2_O/Ag nanocomposites were successfully prepared with the molar ratios of *n*_AgNO3_:*n*_Cu(NO3)2_ = 1:10 at 50 °C and used to fabricate a sensor (Cu_2_O/Ag/GCE). In order to study the interfacial properties of the electrodes, electrochemical impedance spectroscopy (EIS) experiments were conducted. A typical Nyquist plot consists of a semicircle controlled by the electron transfer process in the high-frequency region and a straight line controlled by the diffusion process in the low-frequency region. The semicircle diameter of the curve reflects the electron transfer resistance (R_et_) at the interface between the electrode material and the electrolyte [36]. Figure 6a shows the Nyquist plots of GCE, Cu_2_O/GCE, Cu_2_O/Ag/GCE in 0.1 M KCl solution containing 5 mM [Fe(CN_6_)^3−^]. It is easy to find that the semicircular diameter of the Cu_2_O/Ag/GCE Nyquist plots is smaller than that of the Cu_2_O/GCE curves, which indicates that the introduction of Ag reduces the propagation resistance between the electrode material and the electrolyte improves the electron transfer rate and is beneficial to improving the electrocatalytic performance to some extent.

The electrochemical properties of the electrodes were studied by cyclic voltammetry (CV). Figure 6b shows CV response of the bare GCE, Cu_2_O/GCE, and Cu_2_O/Ag/GCE in the presence of 1 mM H_2_O_2_ in 0.1 M PBS (pH = 7.2) at scan rate of 100 mV/s. From Figure 6b, it can be seen that the responses of the bare GCE toward the reduction of H_2_O_2_ are quite weak. Cu_2_O/GCE exhibits electrochemical response and the cathodic peak (−0.4~−0.17 V) and anodic peak (−0.17~0.1 V) can be ascribed to electrochemical reactions of conversion of Cu_2_O to CuO (oxidation) and CuO to Cu_2_O (reduction), respectively [22]. The electrode reactions involved in the reduction of H_2_O_2_ by the Cu_2_O/Ag nanocomposites can be proposed as follows [37]:

(1) $${\mathrm{Cu}}_{2}{O\;+\;2\mathrm{OH}}^{-}-\;2e^{-}\to {2\mathrm{CuO}\;+\;H}_{2}O$$

(2) $${2\mathrm{CuO}\;+\;H}_{2}O\;+\;2e^{-}\to {\mathrm{Cu}}_{2}{O\;+\;2\mathrm{OH}}^{-}$$

(3) $$H_{2}O_{2}\;+\;2e^{-}\to 2{\mathrm{OH}}^{-}$$

In comparison, Cu_2_O/Ag/GCE showed much higher current response than Cu_2_O/GCE and bare GCE, which proved the point that the introduction of silver improves the electrochemical properties towards H_2_O_2_ of nanocomposites. The enhanced electrocatalytic activity could be ascribed to the synergistic effect of Cu_2_O and Ag. On the one hand, the appearance of a large number of silver seeds causes the Cu_2_O nanocubes to have a small size of less than 100 nm in the process of synthesis. On the other hand, the introduction of silver could enhance the charge transport channels and accelerate the transfer rate of electrons in the reaction [38]. Meanwhile, the active area of reaction is increased by the combination of silver on the Cu_2_O surface, which is beneficial to the adsorption and reaction of H_2_O_2_.

Figure 6c shows CV curves of Cu_2_O/Ag/GCE in the presence of different concentrations of H_2_O_2_. It is obvious that the reduction currents gradually increased with the increase of the H_2_O_2_ concentrations, indicating the good electrocatalytic activity of Cu_2_O/Ag/GCE toward H_2_O_2_ reduction. To investigate the possible kinetic mechanism, the effect of scan rate on the cathodic current was also investigated. As shown in Figure 6d, with the increasing scan rate from 50 to 150 mV s^−1^, the reduction current increased linearly. Figure S4 shows that the linear relationship between cathodic peak current versus square root of scan rate can be obtained (R^2^ = 0.9898), indicating this process was diffusion-controlled.

It is incontrovertible that the detection potential has much influence on the sensitivity of electrochemical sensors. When choosing the detection potential, the peak voltages in CV (−0.4~−0.2 V vs. Ag/AgCl) is preferred for the best reduction performance for H_2_O_2_, while the interference of possible impurities should be considered. The electroactive impurities such as ascorbic acid and uric acid can also be oxidized under high voltages, making it highly likely that their concurrent presences in real applications will interfere with the detection of H_2_O_2_ [39]. Figure 6e shows the current response at different detection potentials upon the successive addition of 0.1 mM H_2_O_2_. Figure 6f shows the corresponding calibration curves of currents vs. H_2_O_2_ concentrations under different potentials. According to Figure 6e,f, though the sensitivity with −0.2 V is lower than that with −0.3 V and almost the same as that with −0.4 V, the profile is more stable and has less background noise. Therefore, the potential of −0.20 V was chosen as the working potential for the detection of H_2_O_2_.

### 3.3. Linear Range, Detection Limit, and Sensitivity of the Cu_2_O/Ag/GCE for H_2_O_2_ Detection

**Figure 7:**
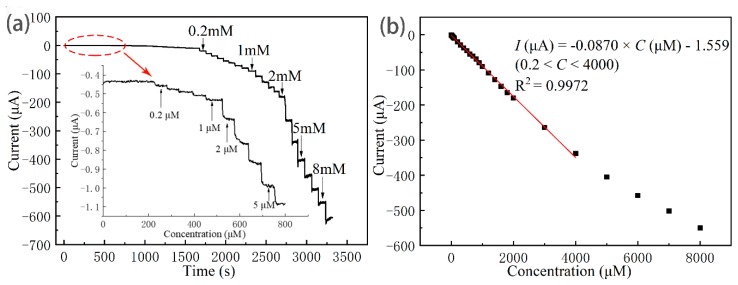
(**a**) Steady-state current–time responses of the Cu_2_O/Ag/GCE upon successive addition of H_2_O_2_ in N_2_-saturated 0.1 M PBS (pH = 7.2) under an applied potential of −0.20 V (vs. Ag/AgCl). Insert: Enlarged image of circle region of (**a**). (**b**) The corresponding calibration curve of currents vs. H_2_O_2_ concentrations. Each dot in (**b**) shows the current value at the corresponding H_2_O_2_ concentration which was obtained in (**a**) and the line is a linear fitting for the experiment points with 0.2 < C < 4000 μM.

The Cu_2_O/Ag nanocomposites-modified electrode was chosen as the sensor electrode for further investigation of H_2_O_2_ sensing for the outstanding electrochemical behavior and the good electrocatalytic reduction performance towards H_2_O_2_ detection. Figure 7a shows the current–time curves of the Cu_2_O/Ag/GCE to the successive addition of H_2_O_2_ into the stirred N_2_-saturated PBS (pH = 7.2) solution at an applied potential of −0.20 V. It can be seen clearly from the enlargement of the current–time curve at low concentrations that the detection limit of Cu_2_O/Ag/GCE for hydrogen peroxide is as low as 0.2 μM (the signal-to-noise ratio of 3, S/N = 3). Figure 7b shows the calibration curve for the H_2_O_2_ sensor, and the linear regression equation was *I* (μA) = −0.0870 *C* (μM) −1.559 with a highly linear relationship (R^2^ = 0.9972), in which *I* is the current and *C* is concentration of H_2_O_2_. Meanwhile, this sensor has a linear detection range from 0.2 to 4000 μM and a sensitivity of 87.0 μA mM^−1^ cm^−2^. In summary, Cu_2_O/Ag/GCE exhibited excellent performance towards the reduction of H_2_O_2_.

Table 1 demonstrates the comparison in the performances of the H_2_O_2_ nonenzyme sensors fabricated based on the use of similar materials as the electrodes in previous literature reports and in this work. It is shown that our Cu_2_O/Ag sensor has a good performance in terms of a high sensitivity, a low detection limit, and a wide linear range. The enhanced electrocatalytic activity could be ascribed to the introduction of silver, which probably provides reaction sites and promotes the electron transfer on the surface of Cu_2_O.

### 3.4. Interference Study

**Figure 8:**
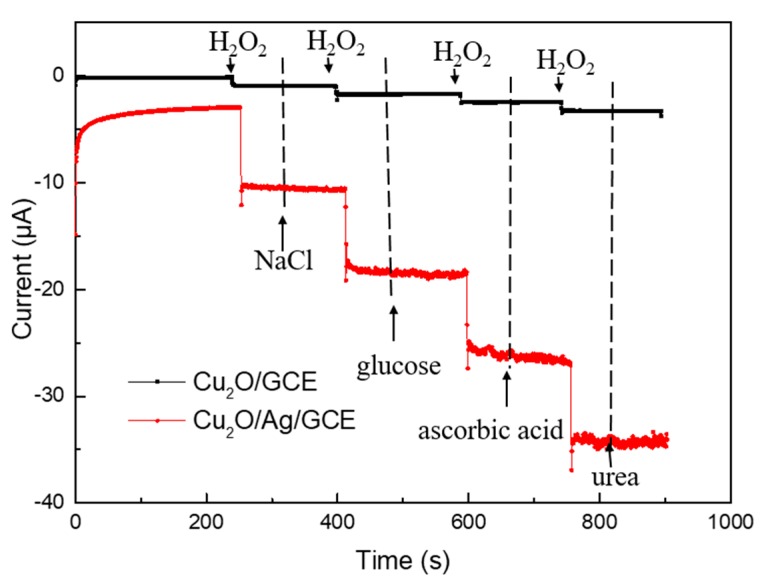
Amperometric response of the Cu_2_O/Ag/GCE and Cu_2_O/GCE successive addition of H_2_O_2_ (0.1 mM), NaCl (1 mM), glucose (1 mM), ascorbic acid (1 mM), and urea (1 mM).

To explore the anti-interference ability of the synthesized Cu_2_O/Ag/GCE (red line) and Cu_2_O/GCE (black line) for H_2_O_2_ detection, we added interfering impurities into a continuous testing system. As shown in Figure 8, between the injections of 0.1 mM H_2_O_2_ solutions, 1 mM NaCl, 1 mM glucose, 1 mM ascorbic acid, and 1 mM urea solutions were added into the 0.1 M PBS solution (pH = 7.2) at −0.20 V in turn. Notably, compared with the Cu_2_O/GCE, the Cu_2_O/Ag/GCE was more sensitive to H_2_O_2_.

The currents for the Cu_2_O/Ag/GCE had obvious changes only when H_2_O_2_ was added. In contrast, the currents did not show any change when the interrupters mentioned above were added. The results indicate that these possible interfering substances do not yield a significant current response, which shows that Cu_2_O/Ag/GCE has a good selectivity for H_2_O_2_.

### 3.5. Reliability and Recovery Test

The reliability test of the Cu_2_O/Ag/GCE was performed by measuring the current response of the electrode upon 1 mM of H_2_O_2_ in 0.1 M PBS solution (pH = 7.2). The average relative standard deviation (RSD) was not more than 4.2%. In a series of eight sensors prepared in the same way, an RSD of 4.8% was obtained, indicating the reliability of this sensor.

To explore the application of the sensor in the practical environment, the recovery test was constructed by adding a certain amount of H_2_O_2_ into milk samples. Before the recovery test experiments were conducted, 5 mL milk purchased from a supermarket was diluted into 50 mL solution using 0.1 M PBS solution first. Then, H_2_O_2_ was added into the as-prepared milk sample with the amounts as shown in Table 2. The results indicate that Cu_2_O/Ag/GCE has the potential to be applied in practical environments.

What we need to be careful about is that the sensors would be better kept in a cool and dry environment to prevent the material from being oxidized in moisture. The service life of the sensor might be improved by using curing materials such as Nafion [36].

## 4. Conclusions

In summary, uniform and small-size Cu_2_O/Ag nanocomposites (size of Cu_2_O particle <100 nm, size of Ag particle <20 nm) were synthesized successfully via a facile one-step process, and successfully used to fabricate an H_2_O_2_ sensor. The electrochemical experiment results reveal that the Cu_2_O/Ag/GCE exhibits outstanding electrochemical behavior and good electrocatalytic reduction performance towards H_2_O_2_. The linear range of the Cu_2_O/Ag/GCE is estimated to be 0.2–4000 μM with a sensitivity of 87.0 μA mM^−1^ cm^−2^ and a low detection limit of 0.2 μM. The anti-interference capability experiment indicated that the Cu_2_O/Ag nanocomposites have good selectivity toward H_2_O_2_. Additionally, the H_2_O_2_ recovery test in the milk solution demonstrates the potential application of Cu_2_O/Ag/GCE in routine H_2_O_2_ analysis.

## Figures and Tables

**Table 1:** The comparison of H_2_O_2_ determination with differently modified electrodes.

Electrode Materials	Detection Potential (V)	Sensitivity (μA mM^−1^ cm^−2^)	Limit of Detection (μM)	Linear Range (μM)	Reference
Porous Cu_2_O	−0.2	50.6	1.5	1.5–1500	[40]
Mesocrystalline Cu_2_O	−0.3	156.6	1.03	2–150	[21]
Graphene/Cu_2_O	−0.4	285	3.3	300–3300	[41]
AgNPs			2.0		[15]
Ag-Au/Cu_2_O	−0.2	4.16	1.3	1.3–1400	[23]
Pt-Cu_2_O/Nafion	−0.25	20.32	10.3	10–6000	[42]
Cu_2_O/Ag	−0.2	87.0	0.2	0.2–4000	This work

**Table 2:** Determination of H_2_O_2_ in milk samples.

Sample	H_2_O_2_ Added (μM)	H_2_O_2_ Found (μM)	Recovery (%)	RSD (%)
1	50	48.4	96.8	1.3
2	100	104.2	104.2	6.1
3	150	142.6	95.1	3.0
4	200	192.4	96.2	1.9

## Supplementary Materials

The Supplementary Materials are available online at https://www.mdpi.com/2079-4991/9/4/523/s1.
