# Supplementary material for: A Facile One-Step Synthesis of Cuprous Oxide/Silver Nanocomposites as Efficient Electrode-Modifying Materials for Nonenzyme Hydrogen Peroxide Sensor
Source: Nanomaterials (Basel). 2019 Apr 3;9(4):523. doi: 10.3390/nano9040523 (PMC6523812; doi:10.3390/nano9040523)
Supplement: Supplementary file 1 [file nanomaterials-09-00523-s001.pdf]

Supplementary Materials

A Facile One-Step Synthesis of Cuprous Oxide/Silver Nanocomposites as Efficient Electrode-Modifying Materials for Nonenzyme Hydrogen Peroxide Sensor

Kaixiang Yang [1,2], Zhengguang Yan [1,2,*], Lin Ma [1,2], Yiping Du [1,2], Bo Peng [1,2] and Jicun Feng [1,2]

1. Institute of Microstructure and Property of Advanced Materials, Beijing University of Technology, Beijing 100124, China; ykx233210@gmail.com (K.Y.); hokingma@gmail.com (L.M.); duyp@emails.bjut.edu.cn (Y.D.); pengbo2017@emails.bjut.edu.cn (B.P.); fengjc0619@163.com (J.F.)
2. Beijing Key Laboratory of Microstructure and Properties of Solids, Beijing University of Technology, Beijing 100124, China

* Correspondence: yanzg@bjut.edu.cn; Tel.: +86-10-67396143

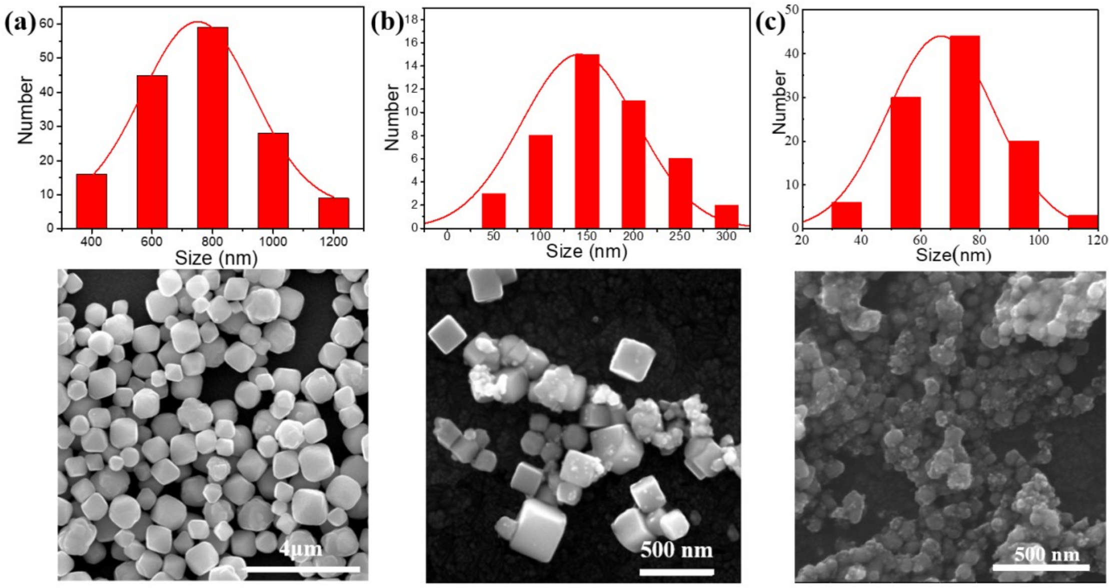

Figure S1 The size distribution histograms of (**a**) pure Cu_2O (400 nm–1.2 μm); (**b**) Cu_2O of nanocomposites (50–300 nm) prepared with n_{AgNO3}:$n_{Cu(NO3)2}$ = 1:20 at 50°C; and (**c**) Cu_2O of nanocomposites (< 100 nm) prepared with n_{AgNO3}:$n_{Cu(NO3)2}$ = 1:10 at 50°C. Note, the SEM images are the same with those in the main text, **Figure 2**.

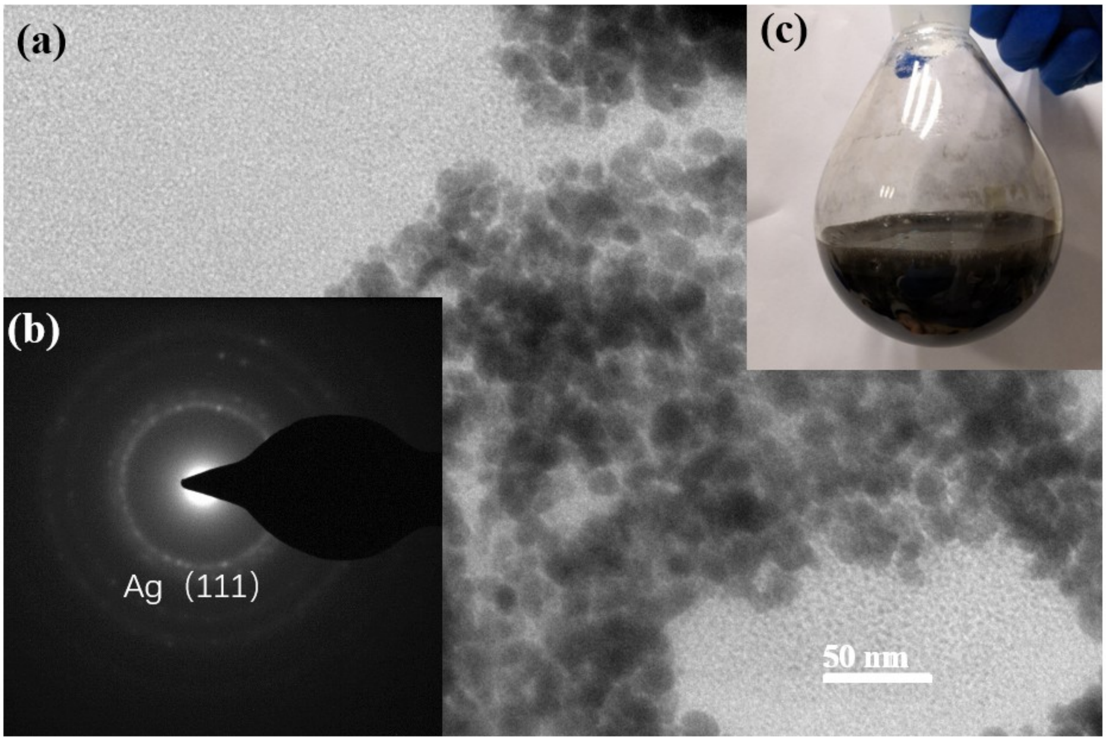

Figure S2 (a) The TEM images of Ag seeds. **(b)** The SAED patterns of Ag seeds. **(c)** The picture of the Ag seeds reaction suspension in a flask. The reaction mixture was added into a flask under stirring of c.a. 500 rpm at room temperature for 10 min and the gray precipitation formed, which is determined as the Ag seeds herein.

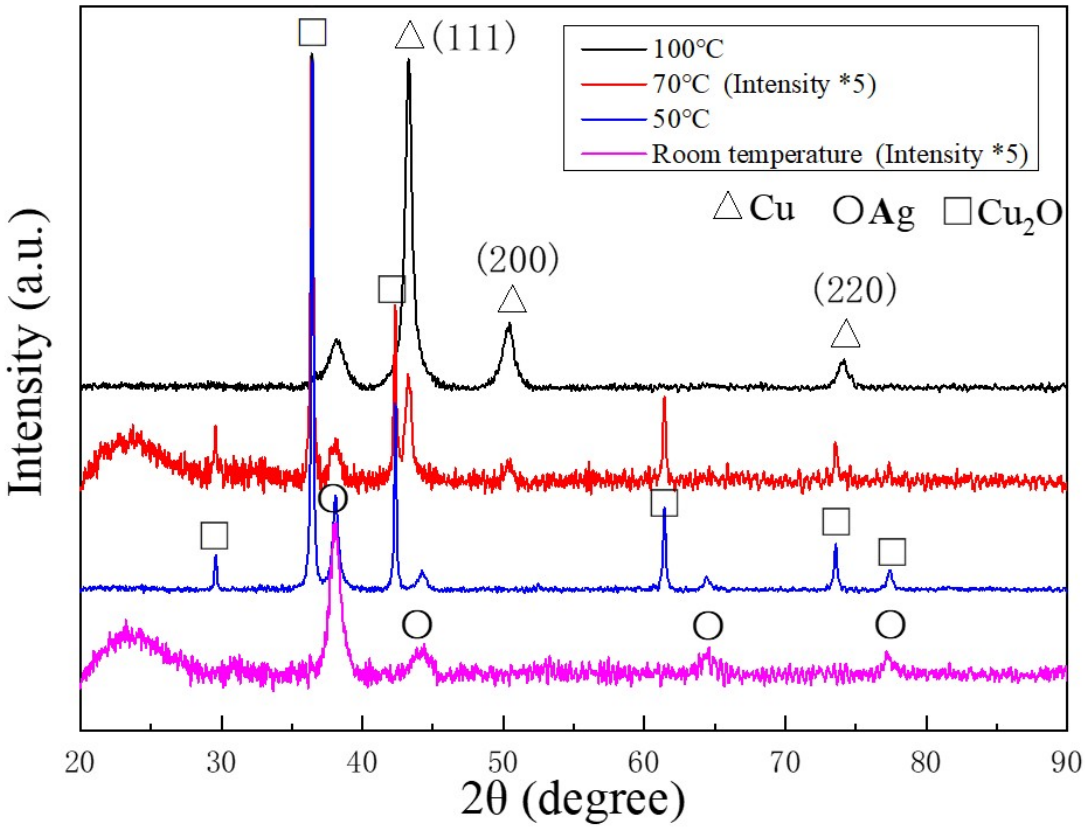

Figure S3 The XRD diffraction patterns of samples prepared with (n_{AgNO3}:$n_{Cu(NO3)2}$ = 1:10) under different temperatures (room temperature, 50 ℃, 70 ℃, 100 ℃, respectively). The Cu peaks (triangle), Ag peaks (round), Cu_2O peaks (square) are labeled. Note, the data for room temperature and 70 ℃ were multiplied with 5 times for a better view. The Cu phase (space group: Fm-3m, JCPDS 65-9026) is with fitted lattice parameter of *a* = 0.36 nm.

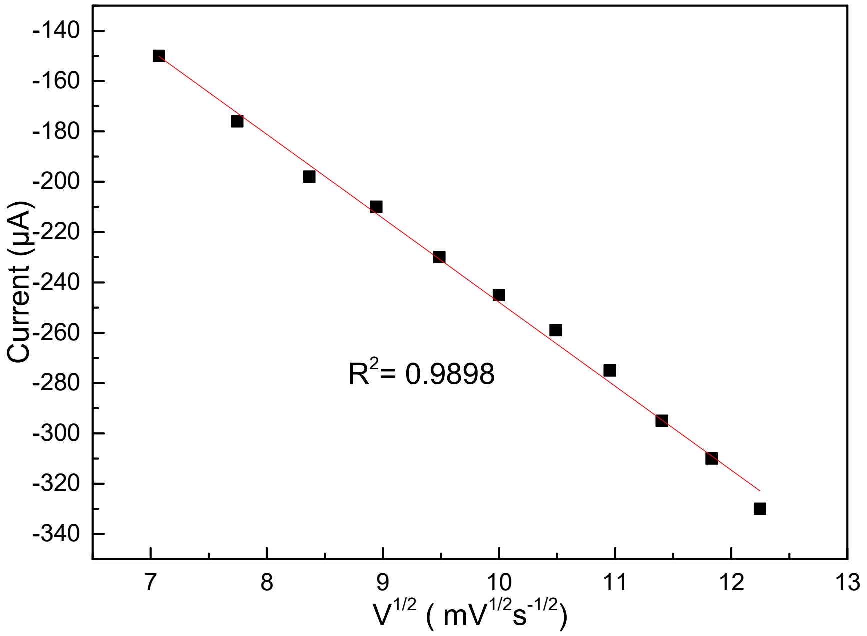

Figure S4 Linear fitting profile of the cathodic peak currents with the square root of scan rate. Also see Figure 6d.
